# Population Health Outcomes of a Student-Led Free Health Clinic for an Underserved Population: A Naturalistic Study

**DOI:** 10.1007/s10900-017-0402-z

**Published:** 2017-07-05

**Authors:** Cynthia M. Stuhlmiller, Barry Tolchard

**Affiliations:** 1 School of Nursing, University at Buffalo, Buffalo, NY USA; 2 School of Health, University of New England, Armidale, NSW 2351 Australia; 3 School of Health & Social Care, Mental Health & Intellectual Disabilities Research Policy Unit, London South Bank University, London, UK

**Keywords:** Population groups, Public health, Student-led clinic, vulnerable populations, Indigenous health

## Abstract

There are a number of hard to reach and underserved communities who experience inadequate health care. In Australia, the Aboriginal and Torres Strait Islanders peoples experience low life expectancy, higher levels for chronic disease and elevated smoking and drinking. These problems are further exacerbated when living in regional and rural Australia and poverty. There are growing concerns over helping such groups in order to close the health disparity gap. A student-led clinic (SLC) was developed to address clinical placement shortages while providing free health and social services in an underserved community in regional Australia. Health data was collected from 2086 attendees enrolled in the SLC to determine health changes and outcomes of student-delivered services. A series of health data was routinely collected at all contact points. This included physical health care, behavioural health risk, and chronic disease measures. All data was recorded in an electronic monitoring system. Population data identified some significant and positive changes to health patterns—smoking, waist size, and body mass index. Unfortunately, gaps in data entry precluded more robust findings. It was clear that this community suffered from experiences commonly associated with health disparity and poverty. There were higher risks of drinking alcohol and smoking with raised levels of lifestyle disease including diabetes. Some of these issues were mitigated by the community being able to attend a locally situated community driven clinic.

## Introduction

There are a number of hard to reach and underserved populations in need of consistent health and social care especially people from indigenous, ethnically diverse and impoverished backgrounds [1]. In Australia, the Aboriginal and Torres Strait Islanders population have experienced high levels of abuse (both structural and interpersonal), poor education, ill health and reduced life expectancies [2]. Today, Aboriginal and Torres Strait Islanders are more likely to drink, smoke and use substances to harmful levels, have higher rates of chronic disease and reduced response to acute health care problems compared to the rest of the Australian population. A number of government approaches have been developed in order to address these issues [3]. Additionally, residents of rural and regional Australia irrespective of indigenous and ethnic diversity experience greater levels of poverty which in turn is associated with harmful behaviours and compromised health outcomes [4]. Identifying community driven approaches that address these health disparities is therefore a priority and alternate healthcare delivery is needed [5].

Student-led health clinics are by no means a new idea [6]. However, with the increasingly fractured health system, rising costs, huge needs of underserved populations, and limited access to care, student dispensed health help may be an increasingly important solution. Additionally, with the shortage of student training placements along with the burden experienced by health professionals in supervising students, the service learning model empowers students to take responsibility for their education as well as figuring out new ways to tackle public health problems. While research indicates that SLCs offer good learning opportunities and satisfactory care with high patient satisfaction levels [7], outcome evaluations have focused on student learning and disease specific parameters, with less emphasis on overall health and lifestyle changes [6, 8].

In 2013, a University student-led clinic (SLC) was developed to address clinical placement shortages while providing free integrated drop in health care in an underserved community with a large Indigenous population. The clinic was developed from an all of community collaboration with local residents, the University and wider health and social care services. This approach to co-production, where everyone worked to establish the services that would meet community need, led to the revitalisation of a community centre in West Tamworth New South Wales Australia [9–11]. This regional area, home to the SLC, serves a population of 3000 people living in a low socio-economic area with high rates of unemployment, high levels of crime and domestic violence, and unmet health needs [12]. These factors combine together to affect the health of individuals and communities. West Tamworth is one of the most highly deprived areas of Australia.

Among other activities, the Centre offers a range of drop-in health services that include primary health, social care, health education, harm minimization, and mental health. Students are responsible for triage, integration of services and direct care in collaboration with other agencies. They also undertake a wide range of health promotion activities, screenings, and health coaching.

The aim of this study was to determine the population health outcomes of clients registered at SLC. Identification of patterns would enable better understanding and focus for targeted practice in order to strengthen student-delivered services.

## Method

This study was given approval by the Universities Institutional Review Board (*HE-13-003*). All subjects signed informed consent to allow their de-identified health data to be used for research. The data collected included demographic information as well as smoking, drinking, weight, height, body mass index, blood pressure, body circumference and, where available, blood values of glucose, cholesterol, and triglycerides. This data was entered into a computerised practice management system known as *Best Practice*. The software program did not allow tracking of individual changes but enabled population data to be retrieved with accuracy.

Data from *Best Practice* was analysed using SPSS version 24. In so doing, it was discovered that not all standard data was being routinely recorded. In most cases *Best Practice* provided excellent throughput information, which was linked to payment for service activity. As part of this analysis of the clinic, these shortfalls soon became obvious leading to a concerted effort by all agencies involved to achieve a minimum data set. Despite this limitation, good population level data was collected over the 18 months of the clinic, which offers insights into the types of health problems and changes such as the number of clients attending who had quit smoking.

## Results

This clinic accepts walk-in members of the community across the lifespan. The average age of attendees was 26 years old with a range from new born to 91 years old. Table 1 provides demographic breakdown by attendees within the previous 12 months and those whom had attended longer than 12 months. From this table it is clear there were even splits on all demographic variables across both time periods.

**Table 1:** Demographics

	<12 months	>12 months
	N (%)	N (%)
Gender		
Female	645 (58.90)	448 (58.11)
Male	450 (41.10)	323 (41.89)
Aboriginal or Torres Strait Islander		
Yes	754 (75.63)	527 (75.07)
No	243 (24.37)	175 (24.93)
Adult or youth		
Youth	425 (48.13)	248 (47.69)
Adult	458 (51.87)	272 (52.31)

### Correlations Between Demographic Variables and Health and Lifestyle Data

Demographic variables were correlated with health and lifestyle measures (Table 2). There were a number of significant relationships using Spearman’s Rho.

**Table 2:** Correlations between demographic variables and health and lifestyle measures

	Gender	ATSI	Adult or youth	Drinks	Smokes	Waist	BMI
Gender	1.000	0.035	−.103**	0.014	0.023	−0.093	0.053
ATSI		1.000	−0.010	.065**	0.001	.282**	.139*
Adult or Youth			1.000	−.286**	−.305**	0.094	.142*
Drinks				1.000	.496**	0.018	−.128*
Smokes					1.000	−.219*	0.040
Waist						1.000	0.409
BMI							1.000

*P < 0.01, **P < 0.001; Spearman’s Rho

These significant correlations reveal more females compared to males in the adult group with the reverse for youth. People who identified as Aboriginal or Torres Strait Islander had higher rates of drinking alcohol. Non-Aboriginal or Torres Strait Islanders had larger mean waist sizes and higher BMIs. Adults had a higher prevalence of smoking and drinking alcohol compared to youth. Adults also recorded higher BMIs compared to youth. People who drank alcohol had higher mean BMI scores than those who did not drink. Smokers had larger mean waist sizes compared to non-smokers.

### Lifestyle Factors—Alcohol Use and Smoking

Among clinic attendees who stated they smoked cigarettes, 59% were light (<11/day), 28% moderate (11–20/day) and 13% heavy smokers (>21/day). Those who drank alcohol reported 52% light (≤10 standard drinks/week), 29% moderate (10–20 standard drinks/week), 9% heavy (21–30 standard drinks/week) and 10% very heavy (30+ standard drinks/week). Associations were tested between demographic variable and these lifestyle choices (smoking and drinking alcohol). There were no associations between gender and drinking or smoking (Fishers exact >0.05). A higher proportion of Aboriginal or Torres Strait Islanders drank alcohol than did non-Aboriginal or Torres Strait Islanders, χ^2^ (1, N = 1337) = 4.08, p = .025. There was no significant association between this variable and smoking. A higher proportion of adults were drinkers than youth, χ^2^ (1, N = 1434) = 115.25, p = .001. There was also a higher proportion of adults who were smokers than youth, χ^2^ (1, N = 1475) = 126.71, p = .001. This is shown in Table 3.

**Table 3:** Proportion of smokers and drinkers by demographic variable

	Smokes			Drinks alcohol		
	Yes	No		Yes	No	
	N (%)	N (%)	OR (95% CI)	N (%)	N (%)	OR (95% CI)
Gender						
Male	242 (25.4)	711 (74.6)	1.11 (0.91–1.34)	200 (21.6)	727 (78.4)	1.05 (0.85–1.30)
Female	308 (27.3)	819 (72.7)		244 (22.4)	846 (77.6)	
Aboriginal or Torres Strait Islander						
Yes	262 (26.4)	731 (73.6)	1.00 (0.82–1.23)	236 (24.4)	733 (75.6)	1.37 (1.10–1.70)
No	250 (26.3)	699 (73.7)		175 (19.1)	742 (80.9)	
Age						
Youth	118 (12.2)	851 (87.8)	0.22 (0.17–0.27)	91 (9.6)	859 (90.4)	0.21 (0.17–0.27)
Adult	414 (38.8)	652 (61.2)		340 (33.2)	683 (66.8)	

Further associations were sought for individuals who may be both smokers and drinkers. Overall, someone who drank alcohol was 12 times more likely to smoke than non-drinkers. Strong associations were found in gender with both female and male smokers being about 5 (relative risk score) times at greater risk of being drinkers than non-smokers. People whom identified as Aboriginal or Torres Strait Islander were 4.5 (relative risk score) times more likely to drink if they smoked compared with non-smokers, and non-Aboriginal or Torres Strait Islanders 5 (relative risk score) times more likely to drink and smoke compared to non-smokers. Finally, youth were 15 (relative risk score) times more likely to drink if they were smokers compared to non-smokers and adults were 5 (relative risk score) times more likely to drink if they were smokers compared to non-smokers. All associations were significant (Table 4).

**Table 4:** Odds ratios and chi square calculations for risk of drinking and smoking combined

	Smokes				
	Drinks alcohol	Yes	No	OR (95% CI)	χ^2^
		N (%)	N (%)		
All					
	Yes	289 (65.1)	155 (34.9)	11.99 (9.40–15.29)	(1, 2012) = 493.97, p = .001
	No	211 (13.5)	1357 (86.5)		
Gender					
Male	Yes	132 (66.0)	68 (34.0)	13.03 (9.05–18.75)	(1, 925) = 238.80, p = .001
	No	94 (13.0)	631 (87.0)		
Female	Yes	157 (64.3)	87 (35.7)	11.20 (8.08–15.53)	(1, 1087) = 255.62, p = .001
	No	117 (13.9)	726 (86.1)		
Aboriginal or Torres Strait Islander					
Yes	Yes	145 (61.4)	91 (38.6)	10.02 (7.16–14.03)	(1, 965) = 214.35, p = .001
	No	100 (13.7)	629 (86.3)		
No	Yes	124 (70.9)	51 (29.1)	15.63 (10.60–23.05)	(1, 918) = 252.96, p = .001
	No	100 (13.5)	643 (86.5)		
Age					
Youth	Yes	65 (70.7)	27 (29.3)	53.29 (30.54–92.98)	(1, 948) = 380.64, p = .001
	No	37 (4.3)	819 (95.7)		
Adult	Yes	215 (63.2)	125 (36.8)	5.31 (4.01–7.04)	(1, 1023) = 145.94, p = .001
	No	167 (24.5)	516 (75.5)		

In an attempt to establish some understanding of the potential impact of the clinic over time, these lifestyle measures were compared between those whom had attended over 1 year ago and those within the past year (Table 5). We found there had not been any reduction in total numbers of people who smoked or drank alcohol between the two time periods. In the cohort seen in the past 12 months’ smokers had a relative risk index of 4.28 and those seen over 12 months ago 5.93 of also being drinkers. Therefore, there was slight reduction in relative risk of someone both smoking and drinking alcohol.

**Table 5:** Changes over time in smoking and drinking alcohol behavior

	Smoker				OR (95% CI)	χ^2^	
	Yes		No				
	N	(%)	N	(%)			
Clinic visit							
<12 months	343	28.0	882	72.0	1.17 (0.95–1.44)	(1, 1951) = 2.185, p = .08	
>12 months	181	24.9	545	75.1			

	Drinking				OR (95% CI)	χ^2^	
	Yes		No				
	N	(%)	N	(%)			
Clinic visit							
<12 months	285	21.9	919	78.1	0.94 (0.75–1.17)	(1, 1888) = 0.34, p = .30	
>12 months	164	23.1	547	76.9			

	Drinks alcohol	Smoker				OR (95% CI)	χ^2^
		Yes		No			
		N	(%)	N	(%)		
<12 months	Yes	168	65.1	90	34.9	10.38 (7.59–14.19)	(1, 1176) = 259.06, p = .001
	No	140	15.3	778	84.7		
>12 months	Yes	107	65.2	57	34.8	15.17 (9.98–23.06)	(1, 545) = 205.93, p = .001
	No	60	11.0	485	89.0		

### Sedentary Lifestyle—Waist Size and BMI

Other related measures of lifestyle included waist circumference and overall BMI. When comparing these variables across demographics, a number of significant differences emerged. There was a significant difference between gender and BMI, with women (M = 23.43) having lower BMIs compared to men (M = 33.35) [*F*(1, 149) = 15.45, p = .006]. There were significant differences in Aboriginal or Torres Strait Islander status for both waist and BMI with non-Aboriginal or Torres Strait Islanders having larger waist sizes [*F*(1, 135) = 6.68, p = .011] and higher BMIs [F(1, 281) = 3.94, p = .048]. This is not surprising giving BMI and waist sizes are measures of potential obesity. However, waist is also a clear marker of potential physical problems including coronary heart disease and type II diabetes. However, that the trend here was for non-Aboriginal subjects to have higher mean scores does not reflect the national picture. These were measures that were not routinely recorded and so results are based on relatively low numbers.

Examining relationships between waist and BMI with smoking and drinking revealed significant results. Smokers (M = 114.66 cm) had significantly larger waist sizes than non-smokers (M = 101.06 cm) [*F*(1, 109) = 6.62, p = .034]. This may reflect a less active lifestyle. However, smokers (M = 26.13) had lower BMIs compared to non-smokers (M = 32.38) [F(1, 242) = 3.25, p = .07 trend]. This may be due to the effect of smoking potentially being an appetite suppressant. There were no differences in those who drank alcohol or for age. There was a significant difference in BMI but not waist size across the two time frames, with those attending the clinic more recently having lower BMIs [*F*(1, 270) = 8.99, p = .003]. In this time, activities had been introduced including a daily walking group and yoga. While these cannot be directly attributed to people having lower BMIs, this is certainly an area for further examination.

### Chronic Conditions

People presented to the SLC with a range of health concerns. Most were attending for short term acute primary care issues such as virus and bacterial infections. Those who attended for chronic conditions fell into seven groups (Table 6). This only accounted for 13.3% of visits. While many had only acute problems, there were issues of inadequate coding, where some diagnoses were not recorded. Therefore, any inferences made with this group need to be supported in a second round of analyses where this problem is being addressed. However, it is reasonable to assume these numbers are fairly representative of the clinic population. In order to increase the power of the analysis multiple imputation methods were used to create a larger sample size. We then performed crosstabs to determine the relationship between the different chronic conditions and lifestyle measures.

**Table 6:** Presentation of chronic health conditions

	N (%)
Attention deficit hyperactivity disorder (ADHD)	226 (2.1)
Mental health	1679 (15.8)
Pulmonary	3311 (31.2)
Chronic heart disease	363 (3.4)
Diabetes	3086 (29.1)
Hypertension	1549 (14.6)
Other	392 (3.7)

Mental health, pulmonary disease, diabetes and hypertension were the main conditions presenting to the clinic. As this is a primary care service, the majority of those presenting with mental health were experiencing either anxiety or depression. Overall, more women (61.0%) experience chronic health conditions than men (39.0%). This was consistent across all conditions except ADHD where males (57.1%) were higher than females (42.9%). Generally, adults experienced chronic conditions more than youth. This would be expected given the nature of such presentations. However, there were significantly higher levels of certain conditions in the youth category including diabetes (30.1% compared to <1% expected), hypertension (13.8% compared to 3.4% expected). In all cases females under 18 experienced higher rates of these conditions. One initiative of the clinic for adults was to provide 24-h blood sugar readings which were recorded and mapped and presented back to residents of the clinic to help them identify their patterns of abnormal readings and the activities they were doing. This provides an immediate feedback system to their behaviours which may be impacting their health with regard diabetes.

### Chronic Condition and Lifestyle

Correlations revealed some chronic conditions were related to drinking alcohol and waists size. Mental Health and ADHD were associated with larger waist sizes. It was interesting to note that people experiencing mental health or pulmonary disease problems were no more likely to be both drinkers and smokers compared with the clinic population as a whole. People with diabetes and hypertension presented with highest proportions of those who both drank and smoked. The conditions with smaller numbers could not have accurate calculations. An association between drinking preference and type of chronic condition was found, χ^2^ (6, N = 7541) = 18.00, p < .01. Examination of the column proportions using the Bonferroni method showed that those with mental health problems were significantly more likely to be drinkers and those with hypertension less (p < .05). No such differences were noted in any other chronic disease group. An association between smoking preference and type of chronic condition was also found, [χ^2^ (6, N = 7766) = 24.53, p < .001]. Those with ADHD were significantly less likely to be smokers while people with diabetes were significantly more likely (p < .05). No such differences were noted in any other chronic disease group (Table 7).

**Table 7:** Relationship between chronic condition and smoking and drinking alcohol

	Alcohol use		Smoker	
	Drinker	Non-drinker	Smoker	Non-smoker
	N (%)	N (%)	N (%)	N (%)
Major condition				
ADHD	28 (17.50)	132 (82.50)	23 (13.77)	144 (86.23)*
Mental health	315 (26.27)*	884 (73.73)	338 (27.04)	912 (72.96)
Pulmonary	555 (23.40)	1817 (76.60)	639 (26.08)	1811 (73.92)
CHD	51 (20.08)	203 (79.92)	64 (24.15)	201 (75.85)
Diabetes	509 (23.26)	1679 (76.74)	640 (28.60)*	1598 (71.40)
Other	77 (28.31)	195 (71.69)	84 (30.22)	194 (69.78)
Hypertension	228 (20.80)	868 (79.20)*	273 (24.42)	845 (75.58)

*Tests are adjusted for all pairwise comparisons within a row of each innermost sub table using the Bonferroni correction (p < .05)

The relative risks of people with chronic health conditions and combined smokers and drinkers were raised across all conditions (Table 8).

**Table 8:** Relationship to both drinking alcohol and smoking with chronic condition

	Drinks alcohol	Smoker				OR (95% CI)	χ^2^
		Yes		No			
		N	(%)	N	(%)		
ADHD							
	Yes	10	35.7	18	64.3	6.67 (2.44–18.25)	(1, 158) = 16.36, p = .001
	No	10	7.7	120	92.3		
Mental health							
	Yes	202	64.1	113	35.9	13.47 (9.89–18.34)	(1, 1194) = 334.90, p = .001
	No	103	11.7	776	88.3		
Pulmonary disease							
	Yes	352	63.4	203	36.6	12.62 (10.10–15.78)	(1, 2368) = 612.16, p = .001
	No	219	12.1	1594	87.9		
CHD							
	Yes	24	47.1	27	52.9	5.33 (2.71–10.49)	(1, 254) = 26.51, p = .001
	No	29	14.3	174	85.7		
Diabetes							
	Yes	345	67.8	164	32.2	11.42 (9.09–14.34)	(1, 2180) = 530.63, p = .001
	No	260	15.6	1411	84.4		
Hypertension							
	Yes	125	54.8	103	45.2	7.01 (5.08–9.66)	(1, 1095) = 163.07, p = .001
	No	128	14.8	739	85.2		

### Chronic Physical Problems

There were related medical problems associated with the chronic health problems identified above. The under reporting of these problems precludes much in the way of meaningful analysis. However, it is useful to understand the general trends of those visiting the clinic in terms of such problems. Two major medical issues were identified; renal problems (N = 50) and Vitamin D deficiency (N = 37), both of which reflect the nature of the deprivation related to population especially dietary issues. Two initiatives of the clinic were a weekly soup kitchen providing residents with at least one nutritionally balanced meal and healthy fruit and vegetables each afternoon after school for children.

While not reported here, case study data indicated a reversal of life threatening and chronic disease conditions, overall improved health parameters, better adherence to treatments, increased health literacy, and lifestyle changes among clients of the SLC. Immediate access to help and cost savings to the health service has been considerable [18].

## Discussion

This paper described the health benefits of a student-led clinic in an underserved community in regional Australia. This community was not representative of the Australian population as whole as there were significantly more people who identified as Aboriginal or Torres Strait Islander. The community was also one of the poorest in Australia. These factors contributed to a population who had high rates of chronic disease, excessive risky behaviours and low health care utilisation related to lifestyle problems.

The risks of drinking alcohol and smoking were increased across the population in prevalence, frequency and severity of use. Smoking appeared to be associated with the community as whole and may have reflected the relative poverty of the area. However, those who identified as Aboriginal or Torres Strait Islander had increased risk of harmful drinking compared with those who did not identify. This is in line with national statistics which highlights higher severity drinking in this group but lower drinking rates overall. Being both a drinker and a smoker were a significant risk for genders, identifying as Aboriginal or Torres Strait Islander and being younger. Drinking is a cultural issue across all walks of Australian life and there is little distinction between men and women partaking in alcohol. In a regional setting, the local hotel (pub) is often the sole source of entertainment and so can become the centre of family nights out. However, despite a smoking ban in hotels, the relationship between smoking and drinking remains a problem in this community. Many hotels find ways around the ban by having open walled rooms considered to be outside or beer gardens close to the bar. In line with national figures the increased risk for Aboriginal or Torres Strait Islanders for being both drinkers and smokers was not unexpected. The increased rates of youth drinking were also in line with national statistics. It has been shown that rural/regional youth drink at much higher rates than urban youth. This is also confounded by parents in rural/regional areas being more encouraging of alcohol use [13].

A function of the clinic was to encourage healthy lifestyle and as such the small change in risk of drinking and smoking over the history of the clinic appeared to show a positive change in attitudes to these behaviours. Activities provided by the students included person cantered smoking cessation programmes, alternative activities for youth including rapping and sports. However, with the exception of those attending the diabetes specialist little was done to target drinking directly. In this service students would provide information and education regarding the harms alcohol causes.

There is a strong association with poverty and deprivation with sedentary lifestyle diseases [14]. In the SLC population we found BMI scores were higher in men compared to women. This was similar to the finding of a large lifestyle survey in Australia [15]. The difference in BMI with Aboriginal or Torres Strait Islanders was unexpected. This was particularly puzzling as this group were overall higher drinkers. The SLC offered healthy eating programmes and education regarding nutrition. However, these were open to all community members. It has been suggested that there be specific ranges in both BMI and waist circumference for Aboriginal and Torres Strait Islanders. Taking these guidelines into account may have produced a different outcome. However, these guidelines have as yet been widely accepted [16].

Chronic health problems are clearly associated with poor lifestyle choices including drinking, smoking and high calorific diets. While the rates of chronic disease, in this population, were not unusual, the higher rates in youth, especially females were worrying. It has been shown that disadvantaged adolescents experience more chronic disease and that this is related to the common risk factors of smoking, drinking and lifestyle. However, education usually reduces the impact of these risks in female adolescents [17]. A number of relational factors emerged between chronic conditions and lifestyle. While mental health and ADHD were associated with larger waist sizes, they were no more likely to be both drinkers and smokers compared with the clinic population as a whole. However, they were at a much higher risk of being a drinker compared with others experiencing chronic conditions. This increased waist in particular may be related to certain medications being used in people with mental health problems. Those experiencing diabetes or hypertension had the highest risk of drinking and smoking. While not reported here, case study data indicated a reversal of life threatening and chronic disease conditions, overall improved health parameters, better adherence to treatments, increased health literacy, and lifestyle changes among clients of the SLC. Immediate access to help and cost savings to the health service has been considerable [18].

It was surprising that diseases of poverty, such as diabetes and hypertension were not significantly higher among Indigenous rather than non-Indigenous clients, given the known higher prevalence of such conditions [19]. This may reflect the poor socio-economic conditions within the area served by the clinic. The high level of diseases of poverty within youth also clearly point to the need for more consistent health checks to ensure chronic health problems do not develop at such a young age. For example, establishing better protocols for recording blood pressure in young people, as well as in adults, is known to be necessary to prevent future problems including stroke and heart failure [20].

## Limitations

It was disappointing to discover the poor level of data entry by students and other health professionals involved in the SLC. Since this data was collected, efforts have been made to improve the quality of the data recorded through closer monitoring. There were some positive changes in the population of clients attending the SLC, including lower waist circumference, BMI, and more clients who had quit or reduced smoking. This may have been attributable to actual client chance, a change in the clientele, or the work of the SLC within the community.

While the positive changes evident at the population level are encouraging, and anecdotal accounts and case studies indicate that the SLC has helped to improve health outcomes, without a more consistent approach to data entry an accurate and comprehensive understanding of these outcomes cannot be achieved. This report signals a call to action to improve data entry practices. Clients, students and clinicians deserve to know the degree to which their collective efforts are improving the health of individuals and the community.
